# ALPPS versus two-stage hepatectomy for colorectal liver metastases—–a comparative retrospective cohort study

**DOI:** 10.1186/s12957-020-01919-3

**Published:** 2020-06-24

**Authors:** Jan Bednarsch, Zoltan Czigany, Samara Sharmeen, Gregory van der Kroft, Pavel Strnad, Tom Florian Ulmer, Peter Isfort, Philipp Bruners, Georg Lurje, Ulf Peter Neumann

**Affiliations:** 1 Department of Surgery and Transplantation, University Hospital RWTH Aachen, Pauwelsstrasse 30, 52074 Aachen, Germany; 2 Department of Medicine III, University Hospital RWTH Aachen, Aachen, Germany; 3 Department of Radiology, University Hospital RWTH Aachen, Aachen, Germany; 4 Department of Surgery, Campus Charité Mitte | Campus Virchow-Klinikum, Charité-Universitätsmedizin Berlin, Berlin, Germany; 5 Department of Surgery, Maastricht University Medical Centre (MUMC), Maastricht, Netherlands

**Keywords:** TSH, ALPPS, CRLM, Oncological outcome

## Abstract

**Abstract:**

**Background:**

Associating liver partition and portal vein ligation for staged hepatectomy (ALPPS) and two stage hepatectomy with inter-stage portal vein embolization (TSH/PVE) are surgical maneuvers applied in patients with advanced malignancies considered unresectable by means of conventional liver surgery. The aim of this report is to compare the oncologic outcome and technical feasibility of ALPPS and TSH/PVE in the scenario of colorectal liver metastases (CRLM).

**Methods:**

All consecutive patients who underwent either ALPPS or TSH/PVE for CRLM between 2011 and 2017 in one hepatobiliary center were analyzed and compared regarding perioperative and long-term oncologic outcome.

**Results:**

A cohort of 58 patients who underwent ALPPS (*n* = 21) or TSH/PVE (*n* = 37) was analyzed. The median overall survival (OS) was 28 months and 34 months after ALPPS and TSH/PVE (*p* = 0.963), respectively. The median recurrence-free survival (RFS) was higher following ALPPS with 19 months than following TSH/PVE with 10 months, but marginally failed to achieve statistical significance (*p* = 0.05). There were no differences in morbidity and mortality after stages 1 and 2. Patients undergoing ALPPS due to insufficient hypertrophy after TSH/PVE (rescue-ALPPS) displayed similar oncologic outcome as patients treated by conventional ALPPS or TSH/PVE (*p* = 0.971).

**Conclusions:**

ALPPS and TSH/PVE show excellent technical feasibility and comparable long-term oncologic outcome in CRLM. Rescue ALPPS appears to be a viable option for patients displaying insufficient hypertrophy after a TSH/PVE approach.

## Background

Colorectal cancer (CRC) is one of the leading causes of tumor-related mortality worldwide with liver metastases being the most common form of distant metastases [1]. In the setting of colorectal liver metastases (CRLM), liver surgery emerged as the mainstay of the curative therapy since it provides excellent oncologic outcome with reported 5-year overall survival (OS) ranging from 16 to 74% [2]. Although liver resection for CRLM is usually considered to be a safe treatment modality with a mortality rate of less than 5% in experienced centers [2], only 15–30% of all patients with CRLM are referred to surgical treatment due to large hepatic tumor burden and/or the presence of multiple extrahepatic metastases [3].

To further improve resectability rates in patients with CRLM, novel surgical procedures such as two-staged hepatectomies combined with inter-stage portal vein embolization (TSH/PVE) and associating liver partition and portal vein ligation for staged hepatectomy (ALPPS) have been added to the armamentarium of the hepatobiliary surgeon in recent years [4, 5]. TSH/PVE has been introduced specifically to address bilateral CRLM [6]. Previous reports from the literature showed comparable survival rates to one-stage surgery as well as an acceptable perioperative mortality but also displayed a significant proportion of patients who do not proceed to stage 2 surgery due to inter-stage tumor progression or insufficient growth of the future liver remnant (FLR) [7, 8]. ALPPS was first reported in 2012 by Schnitzbauer et al., and both the original technique and its novel modifications (partial-ALPPS, etc.) showed a superior and rapid increase of the FLR compared to TSH/PVE, allowing a shorter interval between the first and second step surgeries [4]. Even though the enhanced liver regeneration and volumetric growth triggered by ALPPS might have the potential to overcome the limitations of TSH/PVE, the introduction of ALPPS as a viable option was impeded by a substantially increased perioperative morbidity and mortality [9]. While previous comparisons between TSH/PVE and ALPPS focussed on the perioperative comparability of the procedures, long-term follow-up has been addressed only by a few studies [10–13].

Therefore, the aim of this report is to evaluate the technical feasibility of ALPPS and TSH/PVE and compare the oncologic outcomes of these novel and complex procedures in patients with CRLM.

## Methods

### Patients

Between 2011 and 2017, fifty-eight (*n* = 58) patients with CRLM who were treated with ALPPS or TSH/PVE at our hepatobiliary center were included in this study. Clinical staging was performed according to the Union for International Cancer Control (UICC) recommendations. The study was approved by the Institutional Review Board of the RWTH-Aachen University (EK 341/19) and has been conducted in accordance with the current version of the Declaration of Helsinki, and the good clinical practice guidelines (ICH-GCP).

### Clinical management and surgical technique

All patients underwent a detailed clinical work-up as previously described [14, 15]. In brief, this included a suitable oncological staging by cross-sectional imaging, e.g., contrast-enhanced multiphase computed tomography (CT) and/or dynamic magnetic resonance imaging (MRI) of the liver to visualize metastatic pattern and the relationship of metastases to major anatomic structures.

The individual operative risk was estimated based on the Eastern Cooperative Oncology Group (ECOG)-performance status and the American Society of Anesthesiologists (ASA) classification. Further, liver function determined by laboratory parameters and the quantitative liver function test LiMAx (maximum liver function capacity) and CT or MRI-based prediction of the FLR were incorporated in the operative risk assessment [16]. The decisions for liver resection were made by an experienced hepatobiliary surgeon and approved by the local interdisciplinary tumor board.

ALPPS and TSH/PVE were considered in cases requiring extended liver resections with an estimated FLR of less than 30% in single-step surgery as previously described [14]. Both procedures were also considered in cases with complex oncological situations (e.g., resection of the primary with concomitant “clean up” of one liver lobe) or distinct metastatic patterns which required two-step surgery to safely resect all metastases. Both procedures were technically feasible in all cases except in those patients who underwent rescue ALPPS due to insufficient hypertrophy after an initial TSH/PVE approach. The decision to perform ALPPS vs. TSH in a particular patient was made on a case-by-case basis and surgeon’s preference.

ALPPS was performed according to the recommendations of the latest international consensus conference and as previously described [5, 14]. Briefly, the liver parenchyma was fully or partially transected during stage I using the Cavitron Ultrasonic Surgical Aspirator (CUSA) and intermittent Pringle maneuvers if necessary. The anesthesiologic management was based on restrictive fluid intervention strategy ensuring low central venous pressure (CVP) during actual parenchymal dissection. While portal inflow was dissected to one hemi liver, arterial inflow, bile ducts, and hepatic veins were preserved on both sides to avoid congestion and bile leaks. Volume growth and functional recovery of the liver were ensured by an inter-stage CT scan and a liver function monitoring by LiMAx and standard liver function tests before patients were scheduled for stage II surgery. Here, the hepatectomy was completed [14].

TSH/PVE patients underwent conventional or laparoscopic surgery in stage 1. After recovery from the surgical procedure, these patients did partially undergo PVE as described below and were released from the hospital. If no systemic therapy was conducted prior to stage 1, TSH/PVE patients were scheduled for inter-stage chemotherapy. Stage II was subsequently carried out if liver hypertrophy in the inter-stage CT was sufficient, inter-stage chemotherapy was completed, and no significant tumor progression was observed.

All surgical specimens underwent routine histopathological examination by a trained staff pathologist.

### PVE technique

PVE was carried out using a percutaneous transhepatic ipsilateral approach [16]. In brief, a catheter was inserted into the right portal vein by transhepatic CT-guided puncture of the right portal branch. Embolization of the right portal vein branches (5–8) was performed with a mixture of n-butyl-cyanoacrylate (Braun, Tuttlingen, Germany) and lipiodol (Guerbet, Roissy, France) in a ratio of 1:2 to 1:3. Successful embolization with unrestricted blood flow to the remaining left liver segments was confirmed through repeated portography.

### Volumetric analysis

CT- or MRI-based volumetric analysis was carried out by a senior HPB fellow using a dedicated software (IntelliSpace Portal 8.0 software, Philips healthcare, Amsterdam, The Netherlands). After manual delineation of margins in every slide, total liver volume (TLV), tumor volume (TV), and FLR were subsequently computed by the software. TV was considered as non-functional liver parenchyma for all calculations. Finally, the calculated FLR (cFLR) was computed by the following formula: cFLR [%] = FLR [ml]/(mTLV [ml] − TV [ml]) × 100 [14].

### Follow-up

Every patient of the cohort underwent a regular oncological follow-up after surgery. The follow-up was usually conducted by the referring oncologist or the local outpatient clinic and comprised routine clinical examinations, standard blood tests including up tumor markers (CEA) as well as radiologic cross-sectional imaging. If tumor recurrence was suspected during follow-up, the diagnosis was confirmed by an additional imaging and/or biopsy.

### Statistical analysis

The primary and secondary endpoints in this study were OS and recurrence-free survival (RFS), respectively. OS was defined from the date of the second liver resection to the date of death from any cause or the last contact if the patient was alive. If the patient did not proceed to the second stage of surgery, OS measured from the date of the first liver resection to the date of death from any cause or the last contact if the patient was alive. RFS was defined as the period from surgery to the first recurrence. Categorical data are presented as numbers and percentages and are statistically analyzed using the chi-squared test, Fisher’s exact test, or linear-by-linear association in accordance to scale and number of cases. Continuous variables are presented as median and interquartile range and compared by the Mann-Whitney *U* test. The Kaplan-Meier method was used to estimate survival and the log-rank test to compare distinct survival curves. The associations of OS with clinico-pathological characteristics were determined using univariate and multivariable Cox regression analyses in a forward selection model. Survival curves were generated by the Kaplan-Meier method and compared with the log-rank test. The level of significance was set to *p* < 0.05, and *p* values are given for two-sided testing. Median follow-up was calculated with the reverse Kaplan-Meier method. Analyses were carried out using SPSS Statistics 24 (IBM Corp., Armonk, NY, USA).

## Results

A total of 58 patients underwent either ALPPS (*n* = 21) or TSH/PVE (*n* = 37) for CRLM at our institution from 2011 to 2017. A subset of the ALPPS cohort (*n* = 6, 29%) was initially scheduled for TSH/PVE but underwent rescue ALPPS due to insufficient hypertrophy.

### Preoperative, operative, and postoperative data

The cohort comprises 33 male and 25 female patients with a median age of 60 years. Despite statistically more nodal positive primary tumors in the TSH/PVE (94%, 30/32) than in the ALPPS cohort (57%, 12/21, *p* = 0.001), no statistical difference with respect to the primary tumor in terms of timing (*p* = 0.104), site (*p* = 0.145), and T category (*p* = 0.581) were observed. However, preoperative chemotherapy was more common in the ALPPS (95%, 20/21) than in the TSH/PVE group (57%, 21/37, *p* = 0.038). No statistically significant differences were observed between ALPPS and TSH/PVE regarding the number of cycles of chemotherapy if chemotherapy was applied preoperatively (*p* = 0.243), chemotherapeutic substances applied in first- and second-line chemotherapy (*p* = 0.312, *p* = 0.788), utilization (*p* = 0.128) nor selection of antibodies (*p* = 0.476). Also, no difference in radiological response to preoperative chemotherapy was observed between the groups (*p* = 0.794). The number of lesions (*p* = 0.686) and the largest tumor diameter (*p* = 0.237) at hepatectomy were similar between the groups. More details and group comparisons regarding patients’ demographics and oncologic characteristics are presented in Table 1.

**Table 1:** Patients’ characteristics and oncological data

Variables	ALPPS vs. TSH/PVE analysis		
	ALPPS (***n*** = 21)	TSH/PVE (***n*** = 37)	***p*** value
Demographics			
Sex, m/f (%)	8 (38)/13 (62)	25 (68)/12 (32)	**.029**
Age (years)	60 (52–71)	60 (52–67)	.686
BMI (kg/m^2^)	24 (20–30)	25 (23–29)	.340
ASA, n (%)			.460
I	2 (10)	1 (3)	
II	5 (24)	12 (42)	
III	14 (67)	24 (65)	
Rescue ALPPS after failed TSH, *n* (%)	6 (29)	n.a.	n.a.
Oncologic characteristics			
Timing: synchronous/metachronous	9 (43)/12 (57)	24 (65)/13 (35)	.104
Site of primary			.145
Right-sided colon	6 (29)	4 (11)	
Left-sided colon	10 (48)	17 (46)	
Rectum	5 (24)	16 (43)	
T category primary			.581
I/II	3 (14)	3 (9)	
III/IV	18 (86)	29 (91)	
N category primary			**.001**
N0	9 (43)	2 (6)	
N1	12 (57)	30 (94)	
Preoperative chemotherapy (y/n)	20 (95)/1 (5)	21 (57)/16 (43)	
Number of chemotherapy lines, *n* (%)			**.038**
First-line treatment	20 (95)	27 (73)	
Second-line treatment	3 (14)	7 (19)	
Number of cycles of chemotherapy	8 (6–13)	6 (0–13)	.243
Chemotherapeutic substance in first line, *n* (%)			.312
Oxaliplatin	13 (62)	14 (38)	
Irinotecan	4 (19)	2 (5)	
Oxaliplatin and Irinotecan	2 (10)	6 (16)	
Other/unknown	2 (10)	5 (14)	
Chemotherapeutic substance in second line, *n* (%)			.788
Oxaliplatin	0	1 (14)	
Irinotecan	1 (33)	3 (43)	
Oxaliplatin and Irinotecan	2 (67)	2 (29)	
Other	0	1 (14)	
Antibody utilization, *n* (%)	14 (67)	17 (46)	.128
Antibody, *n* (%)			.476
Bevacizumab	7 (50)	12 (71)	
Cetuximab	5 (36)	4 (24)	
Panitumumab	2 (14)	1 (6)	
Response to chemotherapy, *n* (%)^#^			.794
Stable disease	9 (45)	13 (38.2)	
Partial remission	10 (50)	20 (58.8)	
Progressive disease	1 (5)	1 (2.9)	
Number of lesions at hepatectomy*	3 (1–9)	3 (1–8)	.686
Largest tumor diameter at hepatectomy (mm)*	42 (10–130)	33 (4–107)	.237
Liver function and clinical chemistry			
Albumin (g/l)	41 (34–44)	41 (39–46)	.279
AST (U/l)	32 (27–57)	31 (24–41)	.189
ALT (U/l)	32 (22–47)	27 (20–38)	.197
GGT (U/l)	109 (47–316)	67 (34–173)	.080
Total bilirubin (mg/dl)	0.4 (0.2–0.6)	0.4 (0.3–0.6)	.336
Platelet count (/nl)	254 (196–295)	257 (199–295)	.986
Alkaline Phosphatase (U/l)	148 (91–189)	92 (76–131)	**.029**
Prothrombin time (%)	100 (84–104)	100 (93–111)	.200
Hemoglobin (g/dl)	11.9 (9.9–13.1)	13.2 (12.4–14.5)	**.003**

*ALPPS* associating liver partition and portal vein ligation for staged hepatectomy, *ALT* alanine aminotransferase, *AP* alkaline phosphatase, *ASA* American Society of Anesthesiologists classification, *AST* aspartate aminotransferase, *BMI* body mass index, *CRP* c-reactive protein, *GGT* gamma glutamyltransferase, *INR* international normalized ratio

^*#*^For TSH/PVE patients who did not undergo preoperative chemotherapy, inter-stage chemotherapy was evaluated to determine response to systemic therapy. Data presented as median and interquartile range if not noted otherwise

*Median and range. Categorical data were compared using the chi-squared test, fisher’s exact test or linear-by-linear association according to scale and number of cases. Data derived from continuous variables of different groups were compared by Mann-Whitney *U* Test

A variety of operative procedures was carried out as stage 1 surgery in both groups (Table 2). The median time interval between the stages was significantly longer in the TSH/PVE (57 (34–113) days) than in the ALPPS group (10 (8–14) days, *p* < 0.001). Inter-stage PVE (27/37 vs. 0/21, *p* < 0.001) and inter-stage chemotherapy (9/37 vs. 0/21, *p* = 0.014) was common in TSH/PVE group but was not used in ALPPS patients. Among all patients who underwent stage 1 surgery, 95% (20/21) of the ALPPS patients and 92% of the TSH/PVE patients (34/37, *p* = 0.629) were referred to stage 2 surgery with insufficient hypertrophy being the leading cause of non-completion in both groups. The final R0 rate was comparable in both groups (100% (20/20) vs. 88% (30/34), *p* = 0.116). No perioperative mortality after stage 1 was observed. After stage 2, in-house mortality was 10% (2/20) after ALPPS and 6% (2/34, *p* = 0.577) after TSH/PVE. When stratified for ALPPS, rescue ALPPS, and TSH/PVE, major morbidity (Clavien-Dindo ≥ 3a) occurred in 50% (3/6) of the rescue ALPPS, 71% (10/14) of the ALPPS, and 38% (13/34) of the TSH/PVE patients (*p* = 0.300). The corresponding mortality rates were 14% (2/14) for the ALLPS patients, 6% (2/34) for the TSH/PVE patients, and no mortality was observed in the rescue ALPPS patients (0/6; *p* = 0.458). More operative details and postoperative outcomes are presented in Table 2.

**Table 2:** Operative and postoperative data

Variables	ALPPS vs. TSH/PVE analysis		
	ALPPS (***n*** = 21)	TSH/PVE (***n*** = 37)	***p*** value
Operative data step 1			
Operative time step 1 (minutes)	270 (215–303)	195 (145–250)	**.001**
Operative procedure step 1, *n* (%)			n.a.
Left atypical	0	23 (62)	
Left lateral resection	0	7 (19)	
Right atypical	0	2 (5)	
Right posterior resection	0	1 (3)	
Right hepatectomy	6 (29)	2 (5)	
Right hepatectomy plus atypical left	4 (19)	0	
Right hepatectomy plus left lateral resection	1 (5)	0	
Extended right hepatectomy	3 (14)	0	
Extended right plus atypical left	2 (10)	0	
Right trisectionectomy	4 (19)	0	
Right trisectionectomy plus atypical left	1 (5)	0	
Others	0	2 (5)	
Intraoperative blood transfusion step 1 (y/n)	7 (33)/14 (67)	6 (16)/31 (84)	.133
Inter-stage data			
Time interval between the stages (days)	10 (8–14)	57 (34–113)	**< .001**
Inter-stage chemotherapy (y/n)	0	9 (24)	**.014**
Inter-stage PVE (y/n)	0	27 (73)	**< .001**
Rate of completion, *n* (%)	20 (95)	34 (92)	.629
Reason for non-completion, *n* (%)			.323
Insufficient hypertrophy	1 (100)	2 (67)	
Progressive disease	0	1 (33)	
Operative data step 2			
Operative time step 2 (minutes)	123 (94–170)	280 (240–319)	**< .001**
Operative procedure step 2, *n* (%)			
ALPPS completion	20 (100)	0	
Monosegment resection	0	1 (3)	
Left atypical	0	1 (3)	
Left hepatectomy	0	1 (3)	
Extended left hepatectomy	0	1 (3)	
Right atypical	0	2 (6)	
Right posterior resection	0	2 (6)	
Right hepatectomy	0	17 (50)	
Extended right hepatectomy	0	7 (21)	
Right trisectionectomy	0	2 (6)	
Intraoperative blood transfusion step 2 (y/n)	11 (55)/9 (5)	14 (41)/20 (59)	.325
Volumetric data			
Stage 1			
TLV (ml)	1672 (1589–2127)	1574 (1366–1801)	**.046**
TV (ml)	45 (19–342)	23 (13–60)	.077
FLR (ml)	534 (392–716)	450 (378–560)	.364
cFLR (%)	29 (25–37)	29 (25–36)	.869
Stage 2			
TLV (ml)	2083 (1724–2399)	1679 (1406–1775)	**< .001**
TV (ml)	35 (19–103)	26 (13–68)	.208
FLR (ml)	716 (504–954)	655 (486–744)	.448
cFLR (%)	37 (30–47)	31 (33–50)	.103
Degree of hypertrophy (%)	35 (23–62)	45 (18–69)	.803
Postoperative data			
Intensive care step 1, days	1 (1–2)	0 (0–1)	**< .001**
Intensive care step 2, days	1 (0–1)	1 (1–1)	**.044**
Hospitalization step 1 and 2, days	32 (22–46)	22 (17–28)	**.009**
Clavien-Dindo stage 1 ≥ IIIa, *n* (%)	2 (10)	5 (14)	.654
Clavien-Dindo stage 1 = V, *n* (%)	0	0	n.a.
Clavien-Dindo stage 2 ≥ IIIa, *n* (%)	13 (65)	13 (38)	.057
Clavien-Dindo stage 2 = V, *n* (%)	2 (10)	2 (6)	.577
Follow-up data			
R0 resection	20 (100)	30 (88)	.116
Median RFS (95% CI), months	19 (8–30)	10 (6–14)	.050
Median OS (95% CI), months	28 (19–37)	34 (25–43)	.963

Data presented as median and interquartile range if not noted otherwise. Categorical data were compared using the chi-squared test, Fisher’s exact test, or linear-by-linear association according to scale and number of cases. Data derived from continuous variables of different groups were compared by Mann-Whitney *U* test. Oncological survival was compared by the log-rank test. Survival data were calculated under exclusion of perioperative mortality

*ALPPS* associating liver partition and portal vein ligation for staged hepatectomy, *cFLR* calculated future liver remnant, *FLR* future liver remnant, *OS* overall survival, *PVE* portal vein embolization, *RFS* recurrence-free survival, *TLV* total liver volume, *TV* tumor volume

### Survival analysis

**Fig. 1:**
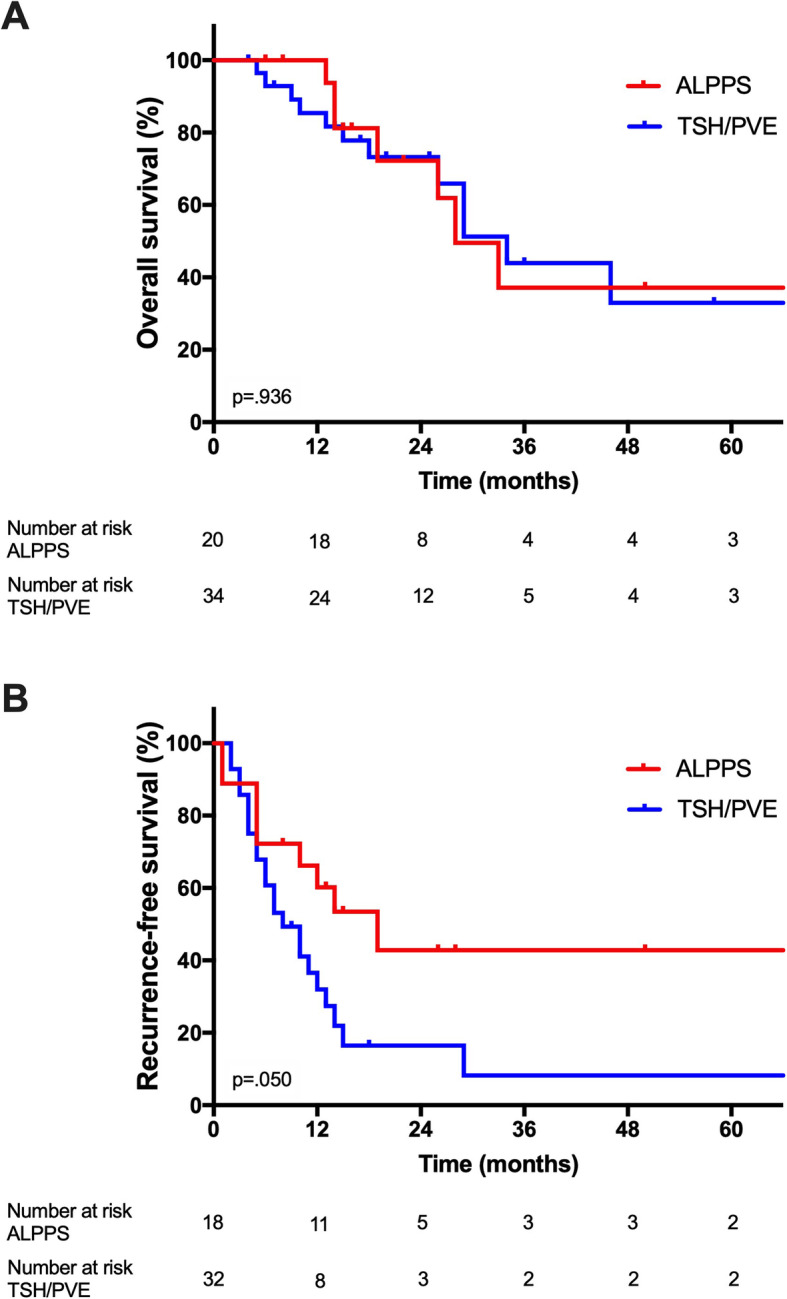
Overall and recurrence-free survival after ALPPS and TSH/PVE. **a** Overall survival. The median OS after ALPPS and TSH/PVE was 2.3 years (95% CI 1.6–3.1) and 2.8 years (95% CI 2.1–3.6), respectively. **b** Recurrence-free survival. The median RFS after ALPPS and TSH/PVE was 1.6 years (95% CI 0.7–2.5) and 0.8 years (95% CI 0.5–1.2), respectively. CI, confidence interval; OS, overall survival; RFS, recurrence-free survival

After a median follow-up of 22 months (0–89 months/range), a total of 24 out of 57 patients of the study cohort have died over the follow-up period. The median OS of the overall cohort was 33 months. The median OS was 34 months after TSH/PVE and 28 months after ALPPS (*p* = 0.297 log rank, Fig. 1a). The corresponding 3-year OS and 5-year OS were 44% and 33% after TSH/PVE and 37% and 37% after ALPPS, respectively. The median RFS was shorter after TSH/PVE (10 months) compared to the ALPPS group (19 months), but the difference did not reach statistical significance (*p* = 0.05 log rank, Fig. 1b). The 3-year RFS and 5-year RFS were 8% and 8% after TSH/PVE, 43% and 43% after ALPPS, respectively.

**Fig. 2:**
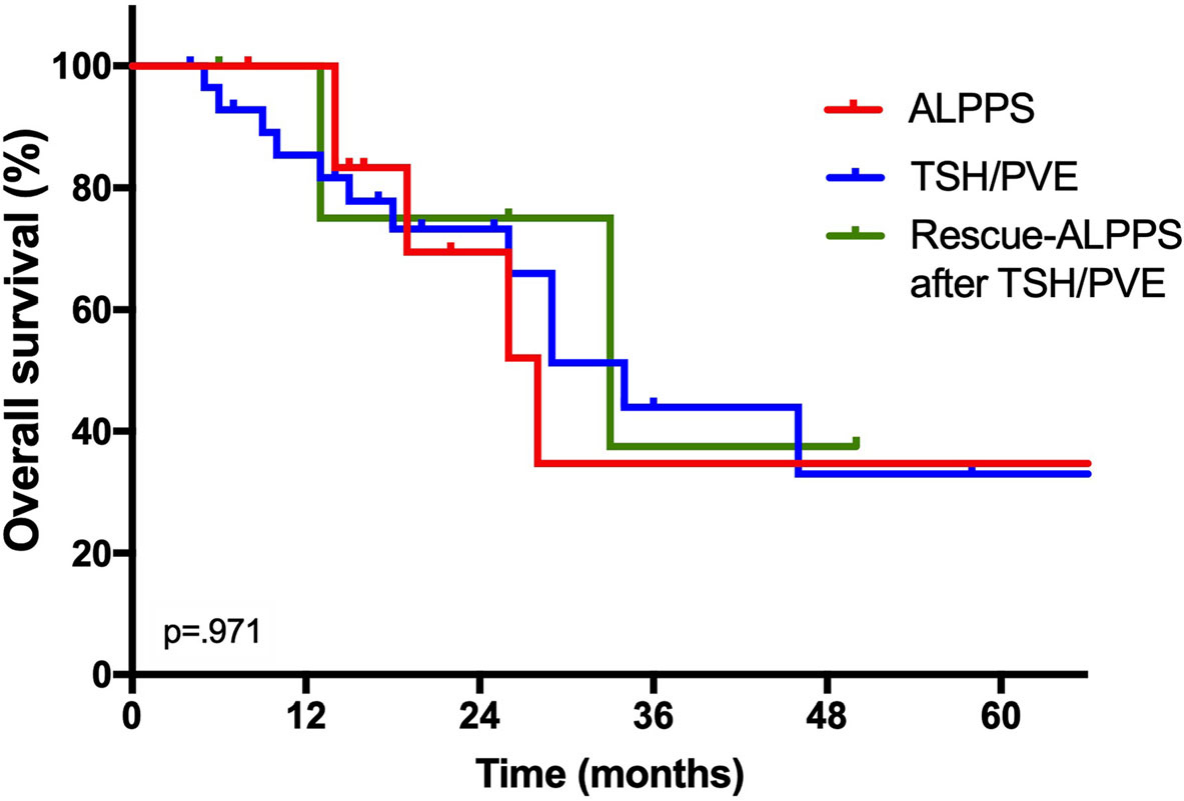
Overall survival after ALPPS, rescue ALPPS, and TSH/PVE. In this sub-analysis, patients who underwent rescue ALPPS due to insufficient hypertrophy after an initial TSH/PVE approach were excluded from the ALPPS group and analyzed separately. Here, no difference in OS after TSH/PVE (2.8 years, 95% CI 2.1–3.6), ALPPS (2.3 years, 95% CI 1.5–3.2), and rescue ALPPS (2.8 years, 95% CI 2.3–3.3, *p* = 0.971, log rank) was observed. CI, confidence interval; OS, overall survival

A secondary analysis excluding patients who underwent rescue ALPPS due to insufficient hypertrophy after an initial TSH/PVE approach from the ALPPS group and analyzing them separately showed no difference in OS after TSH/PVE (34 months), ALPPS (28 months), and rescue ALPPS (33 months, Fig. 2, *p* = 0.971 log rank). Also, no difference was observed regarding RFS after TSH/PVE (10 months), ALPPS (19 months), and rescue ALPPS (19 months, *p* = 0.147 log rank).

Further, a univariate Cox regression analysis was conducted to determine predictors of OS. Here, response to preoperative chemotherapy (*p* = 0.024) and preoperative c-reactive protein (CRP, *p* = 0.045), but not the operative approach (ALPPS vs. TSH/PVE, *p* = 0.954), were associated with OS (Table 3). Variables showing a *p* value < 0.1 were subsequently included in a multivariable Cox regression analysis. Here, response to preoperative chemotherapy (*p* = 0.013) and the number of preoperative cycles of chemotherapy (*p* = 0.024) were independently associated with OS. None of the variables demonstrating a statistical significance in the group comparison between ALPPS and TSH/PVE patients showed significant results for OS in the Cox regression analyses.

**Table 3:** Univariate and multivariable analysis of survival

	Univariate analysis		Multivariate analysis	
	HR (95% CI)	***p*** value	HR (95% CI)	***p*** value
**Demographics**				
Sex (female = 1)	1.27 (0.56–2.92)	.569		
Age (≤ 60 years = 1)	2.18 (0.96–4.93)	.063	excluded	
BMI (≤ 25 kg/m^2^ = 1)	1.30 (0.16–10.33)	.803		
ASA (I/II = 1)	1.47 (0.61–3.56)	.393		
**Oncological data**				
Timing (synchronous = 1)	2.03 (0.90–4.58)	.090	excluded	
Site of primary		.471		
Right-sided colon	1			
Left-sided colon	0.50 (0.17–1.48)			
Rectum	0.67 (0.22–2.03)			
T category primary (T1/T2 = 1)	4.80 (0.63–36.67)	.131		
N category primary (N0 = 1)	0.60 (0.22–1.67)	.330		
Preoperative chemotherapy (no = 1)	1.92 (0.57–6.46)	.292		
Second-line treatment (no = 1)	1.03 (0.38–2.76)	.958		
Number of cycles of chemotherapy		.060		**.024**
≤ 6	1		1	
> 6	3.38 (0.95–12.03)		5.92 (1.26–27.78)	
Antibody utilization (no = 1)	1.29 (0.57–2.90)	.540		
Response to chemotherapy		.**024**		**.013**
Stable disease	1		1	
Partial remission	0.32 (0.13–0.78)		0.17 (0.05–0.55)	
Progressive disease	n. a.		n. a.	
Number of lesions at hepatectomy		.763		
≤ 3	1			
> 3	1.14 (0.48–2.70)			
Largest tumor diameter at hepatectomy		.099	excluded	
≤ 35	1			
> 35	1.98 (0.88–4.44)			
**Clinical chemistry**				
AST (≤ 30 U/l = 1)	0.52 (0.23–1.17)	.112		
ALT (≤ 30 U/l = 1)	0.78 (0.35–1.75)	.550		
GGT (≤ 75 U/l = 1)	1.00 (0.44–2.27)	.944		
Bilirubin (≤ 0.4 mg/dl = 1)	1.54 (0.68–3.48)	.296		
Alkaline phosphatase (≤ 105 U/l = 1)	1.87 (0.81–4.30)	.143		
INR (≤ 1 = 1)	0.77 (0.32–1.85)	.553		
Prothrombin time (≥ 100% = 1)	0.86 (0.37–2.02)	.731		
Hemoglobin (≤ 13 g/dl = 1)	0.77 (0.34–1.73)	.520		
CRP, mg/l (≤ 10 mg/l = 1)	2.38 (1.02–5.57)	**.045**	excluded	
**Operative and inter-stage data**				
Operative approach		.954		
ALPPS	1			
TSH	.976 (0.43–2.23)			
Operative time step 1 (≤ 180 min = 1)	1.02 (0.42–2.45)	.972		
Blood transfusion step 1 (no = 1)	1.18 (0.47–2.98)	.772		
Interval between stages (≤ 35 days = 1)	0.69 (0.28–1.67)	.408		
Inter-stage chemotherapy (no = 1)	0.04 (0.01–4.11)	.171		
Completion (no = 1)	0.46 (0.06–3.67)	.460		
Operative time step 2 (≤ 240 min = 1)	0.83 (0.37–1.86)	.649		
Blood transfusion step 2 (no = 1)	1.66 (0.73–3.78)	.231		
**Pathological data**				
R1 resection (no = 1)	0.04 (0.01–44.68)	475		

Univariate and multivariable Cox regression analyses were carried out to determine predictors of overall survival. Variables displaying a *p* value < 0.1 were included in the multivariable Cox regression model

*ALPPS* associating liver partition and portal vein ligation for staged hepatectomy, *ALT* alanine aminotransferase, *AP* alkaline phosphatase, *ASA* American Society of Anesthesiologists classification, *AST* aspartate aminotransferase, *BMI* body mass index, *CRP* c-reactive protein, *GGT* gamma glutamyltransferase, *INR* international normalized ratio

## Discussion

ALPPS and TSH/PVE are advanced surgical maneuvers applied in liver malignancies which are unresectable by conventional liver surgery. In the context of CRLM, this translates to patients with extensive tumor burden and/or unfavorable tumor biology who would otherwise be referred to palliative systemic therapy [17]. In our series, we analyzed OS after both procedures in a large monocentric cohort and found a 5-year-OS of 37% after TSH/PVE and 33% after ALPPS, respectively. Given the nature of the complex clinical situations where ALPPS or TSH/PVE are required to ensure a complete tumor removal and the alternative of systemic therapy without liver resection, our oncologic outcome supports the utilization of both procedures in CRLM [18, 19].

The primary aim of our study was to compare oncologic outcome of ALPPS and TSH/PVE. We therefore analyzed the distribution of oncologic risk factors in both groups and found no statistical differences in major characteristics despite more nodal positive primary tumors in the TSH/PVE group and a more frequent use of preoperative chemotherapy in the ALPPS group. However, the latter can be balanced by a high rate of inter-stage chemotherapy in case of TSH/PVE. With respect to the equal distribution of oncologic risk factors, we found no significant difference in OS between the two distinct procedures. In contrast, RFS appeared to be better after ALPPS with a median RFS of 19 months compared to 10 months after TSH/PVE, a difference which marginally failed to achieve statistical significance (*p* = 0.05).

Comparative analyses of the two procedures are scarce since most of the previous studies were focusing on perioperative characteristics and provided almost exclusively short-term data or analyzed limited sample sizes in terms of oncological outcome [11, 20, 21]. Therefore, only two studies were comparable to our cohort with respect to statistical approach and sample size [10, 22].

Robles et al. reported their experience with the technical modification “tourniquet-ALPPS” and compared oncologic outcome of these patients with individuals undergoing TSH/PVE in a matched-pair analysis. This study showed no difference in oncologic and perioperative outcomes [22]. In contrast, Adam et al. showed a superior survival after TSH/PVE with a median OS of 37 months compared to 20 months after ALPPS [10]. A possible explanation for this observation might be a high rate of R1 margin in both groups and more R1 resections after ALPPS (14/17) than after TSH/PVE (18/26) in this particular cohort, although the general prognostic ability of margin status in CRLM is a topic of an ongoing debate [23, 24]. Interestingly, a significant proportion of patients who underwent TSH/PVE approach in the study of Adam et al. did not proceed to stage 2 surgery (15/41), and these particular individuals displayed similar long-term outcome compared to the completed ALPPS group. In our analysis, 92% of our patients completed both stages of surgery due to our policy to attempt to “salvage” patients failing to proceed to second stage using a rescue ALPPS procedure [25]. Thus, a subset of patients of our ALPPS group (6/21) were initially scheduled for TSH/PVE but were salvaged by ALPPS after insufficient hypertrophy. As the ALPPS group was stratified in “conventional” ALPPS and rescue ALPPS, no difference in OS was observed after “conventional” ALPPS, rescue ALPPS, and TSH/PVE which further supports the role of ALPPS in cases where TSH/PVE fails.

Of note, the results of the multicentric, randomized Scandinavian LIGRO trial have recently been published [26]. Here, median OS for patients randomized to ALPPS was 46 months compared with 26 months for patients randomized to TSH, while the median RFS was 11 months after ALPPS and 8 months after TSH. Although the results of this randomized trial suggest favoring ALPPS over TSH, it has to be considered that Hasselgren et al. report a large proportion of non-resected patients after TSH (10/49) compared to ALPPS (3/48) as well as a significant amount of rescue ALPPS in the TSH group (12/39). Also, the rate of R1 resections was notable in both ALPPS (10/45) and TSH (12/38) compared to our cohort. Interestingly, individuals undergoing rescue ALPPS performed worse from an oncological point of view compared to actual TSH and ALPPS patients. Other differences between our study and the LIRGO trial can be displayed by looking deeper into patient demographics and the respective Cox regression analyses for independent predictors of OS. While only a small set of 15% of the patients in the LIRGO trial presented with metachronous disease, 43% of our cohort represent patients with metachronous CRLM. Additionally, all individuals enrolled to the LIRGO trial underwent chemotherapy prior to stage 1 surgery in contrast to our study with 71% of the patients undergoing preoperative systemic therapy. The most important predictors of OS in our overall cohort were the number of preoperative cycles of chemotherapy and response to systemic therapy (Table 3). Interestingly, neither of these characteristic features showed any significant hazard ratios in the Cox regression analysis of the LIRGO trial. Instead, the allocated treatment (ALPPS vs. TSH), rate of completion, ASA scoring, largest tumor diameter, and postoperative complications were the best predictors for OS.

Considering this particular difference in oncologic and demographic characteristics, the good results of rescue ALPPS in our cohort, and a larger rate of R1 resections and more TSH failures in the LIRGO trial, the distinctions to our study might be sufficiently explainable. The comparison between our study and the LIRGO trial does further show that the approach to ALPPS and TSH/PVE in the scenario of CRLM is largely different between centers further complicating any valid comparison between various study cohorts.

The perioperative morbidity and mortality in our cohort is in line with previous findings [11, 13, 21, 27, 28]. While no patient deceased after stage 1, operative mortality was 10% for ALPPS and 6% for TSH/PVE after stage 2. Major morbidity defined as higher than Clavien-Dindo II was more common in ALPPS than in TSH/PVE with 64% versus 38% of the patients experiencing relevant complications after step II, but this difference did not reach statistical significance. Of note, in a recent meta-analysis ALPPS was associated with higher mortality compared to TSH/PVE [20]. This particular finding was confirmed neither in our present study nor by Robles et al. [22]. We assume that the technical evaluation and surgical refinement of ALPPS in the last decade and the accumulating experience with the procedure in hepatobiliary centers resulted in better patient selection and more sophisticated clinical decision-making and translated to perioperative outcomes that are more comparable to conventional major liver surgery [29].

A superior kinetic growth rate has always been an argument for ALPPS when compared to TSH/PVE [21, 27]. Interestingly, we were not able to detect differences in the final cumulative FLR prior to stage 2 or in the degree of hypertrophy between the two groups. This might be related due to the relevant percentage of rescue ALPPS patients in our ALPPS cohort. These particular individuals already showed some degree of hypertrophy after the TSH/PVE approach, so the additional parenchymal dissection in stage 1 of ALPPS might result in an inferior hypertrophy response when compared to the conventional ALPPS procedure. Another reason for our results might be the long inter-stage interval in TSH/PVE group. A recent meta-analysis assessed volumetric data of ALPPS and TSH/PVE patients [20]. Here, even though no statistical difference was noted in preoperative FLR and the extent of FLR increase, a shorter period of time was needed to reach the final FLR. There is, however, a considerable heterogeneity among previous reports [20].

Comparability in terms of the oncological disease between two surgical procedures is always a topic of intense discussions in retrospective studies. We observed no statistical differences between the two groups in most of the preoperative oncological characteristics. Timing, site, and T category of the primary tumor as well as number of cycles of chemotherapy, chemotherapeutic substances applied in first- and second-line chemotherapy, utilization, and selection of antibodies were equally distributed between the groups. Also, the number of lesions and the largest tumor diameter at hepatectomy were similar between the groups. However, nodal positive primary tumors were more common in TSH/PVE and preoperative chemotherapy more common in ALPPS patients, respectively (Table 1). While the latter can be balanced by significantly more inter-stage chemotherapy in individuals undergoing TSH/PVE, the unequal distribution of nodal positive primary tumors has to be discussed critically. Lymph node status of the primary tumor is traditionally considered to have an important prognostic role in patients undergoing liver resection for CRLM [30]. This paradigm has been recently challenged by reports questioning the prognostic value in sub cohorts of patients with CRLM [31, 32]. In a Dutch study, lymph node status was predictive for overall survival in rectal but not for colon cancer as primary [31]. In another North American study evaluating the prognostic value of the number of regional lymph node metastases of the primary tumor for survival in patients undergoing resection of CRLM, the predictor lost its prognostic significance in the subset of patients who received perioperative chemotherapy [32]. This study has also important implications for our cohort, since the vast majority of our patients underwent excessive chemotherapy due to generally large tumor burden. Whether the lymph node status of the primary tumor has prognostic ability in the era of modern chemotherapy or not is subject of an ongoing debate and is beyond the scope of our current study. However, our multivariable Cox regression analyses showed no influence of nodal status on OS in the investigated cohort. It should be noted, however, that more than half of our study population (58%, 33/57) presented with synchronous CRLM and therefore underwent preoperative chemotherapy prior to resection of the primary which might limit the validity of lymph node status in our analysis. Nevertheless, a potential effect of lymph node status has to be taken into account when interpreting the data of our present report.

Our analysis has certainly some limitations which have to be discussed critically. Firstly, this study was conducted in a single center based on the authors’ distinct clinical management in TSH and ALPPS in the setting of CRLM. Thus, the results might therefore be not fully transferable to other centers in all details. Secondly, as obvious, our analysis was based on a limited sample size. A larger sample size would have qualified for a propensity-score matching approach, which would have provided a more solid statistical basis to our analysis. Nevertheless, ALPPS is a procedure which is normally restricted to highly selected patients and experienced centers, and available studies addressing the issue comprise a comparable number of patients. Therefore, further investigations in multi-centric setting with a significantly larger sample size are needed to validate our findings. Thirdly, our data was not obtained during a controlled prospective clinical trial, and no strict protocol on which procedure should be preferred in particular metastatic patterns or oncological situations was established during study period. This lack of a randomized controlled design may limit the validity of our findings due to a considerable risk of a significant selection bias. It should be noted, however, that there is currently just one randomized trial on the topic of oncologic outcomes following ALPPS or TSH available in the literature [26]. This shortage of prospective controlled studies further underlines the importance of retrospective analyses to determine the role of ALPPS and TSH/PVE in the oncologic outcome following liver resections in patients with CRLM.

## Conclusion

Considering the discussed limitations, we conclude that ALPPS and TSH/PVE showed excellent technical feasibility and comparable long-term oncologic outcome in CRLM. Moreover, rescue ALPPS appears to be a viable option for patients displaying insufficient hypertrophy after an initial TSH/PVE approach.

## Data Availability

The datasets used and/or analyzed during the current study are available from the corresponding author on reasonable request.

## Abbreviations

ALPPS: Associating liver partition and portal vein ligation for staged hepatectomy
ALT: Alanine aminotransferase
AP: Alkaline phosphatase
ASA: American Society of Anesthesiologists
AST: Aspartate aminotransferase
BMI: Body mass index
CEA: Carcinoembryonic antigen
cFLR: Calculated future liver remnant
CI: Confidence interval
CRC: Colorectal cancer
CRLM: Colorectal liver metastases
CRP: C-reactive protein
CT: Computed tomography
CUSA: Cavitron Ultrasonic Surgical Aspirator
ECOG: Eastern Cooperative Oncology Group
FLR: Future liver remnant
GGT: Gamma glutamyltransferase
INR: International normalized ratio
LiMAx: Maximum liver function capacity
MRI: Magnetic resonance imaging
OS: Overall survival
PVE: Portal vein embolization
RFS: Recurrence-free survival
TLV: Total liver volume
TSH: Two-stage hepatectomy
TV: Tumor volume
UICC: Union for International Cancer Control
